# Physical and Physiological Profiles of Brazilian Jiu-Jitsu Athletes: a Systematic Review

**DOI:** 10.1186/s40798-016-0069-5

**Published:** 2017-02-13

**Authors:** Leonardo Vidal Andreato, Francisco Javier Díaz Lara, Alexandro Andrade, Braulio Henrique Magnani Branco

**Affiliations:** 1 Sciences Center of Health and Sport, State University of Santa Catarina, Florianopolis, Brazil; 2 Faculty of Sport Sciences, Sport Training Laboratory, University of Castilla-La Mancha, Toledo, Spain; 3 School of Physical Education and Sport, University of Sao Paulo, Sao Paulo, Brazil; 4 University Center of Maringa – UNICESUMAR, Maringa, Parana Brazil

**Keywords:** Combat sports, Physical fitness, Physical evaluation

## Abstract

**Background:**

Brazilian jiu-jitsu is a grappling combat sport that has intermittency as its core element; in other words, actions of high, moderate and low intensity are interspersed during matches, requiring a high level of conditioning to support optimal levels of performance for the total match time. The athletes perform from four to six matches during a day of competition, and this number may increase if the open-class competition, which is held parallel to the competition by weight class, is considered. This systematic review examined the physical and physiological profiles of Brazilian jiu-jitsu athletes.

**Methods:**

Only scientific researches dealing with the major fitness components of Brazilian jiu-jitsu athletes (i.e. body composition and somatotype, aerobic and anaerobic profiles, muscular strength and power) and using accepted methods that provided relevant practical applications for a Brazilian jiu-jitsu athlete’s fitness training and/or performance were included in the current review. A computer literature search was carried out of the PubMed, ISI Web of Knowledge, SportDiscus and Scopus databases (up to January 2016).

**Results:**

The database research generated 205 articles. After the application of inclusion and exclusion criteria, 58 studies were included for the present systematic review. A total of 1496 subjects were involved in all the selected investigations.

**Conclusions:**

Body fat is generally low for these athletes and the mesomorphic component is predominant. The different studies showed VO_2max_ values between 42 and 52 mL/kg/min, and it seems that aerobic fitness does not discriminate among Brazilian jiu-jitsu athletes of different competitive levels. There is a lack of scientific studies that have investigated anaerobic responses both in lower and upper limbs. Maximal dynamic, isometric and endurance strength can be associated with sporting success in Brazilian jiu-jitsu athletes. Although decisive actions during Brazilian jiu-jitsu matches are mainly dependent on muscular power, more specific studies are necessary to describe it. Studies involving the female sex should be conducted. In addition, further research is needed to analyse whether there are differences between sex, belt ranks and competitive level, and among the different weight categories for different variables.

## Key Points

- Brazilian jiu-jitsu athletes had low body fat without differences between novices and experts or between elite and non-elite athletes.
- Aerobic power was similar to that of other grappling combat sports and did not seem to be influenced by the Brazilian jiu-jitsu athlete’s competitive level.
- Experience and competitive level seem to influence an athlete’s flexibility responses as experienced athletes had greater flexibility than beginners and elite athletes showed more flexibility than non-elite athletes.

## Background

In the last decade in particular, there has been a significant rise in the popularity of Brazilian jiu-jitsu. Part of this is due to the success of Brazilian jiu-jitsu athletes in mixed martial arts events [1]. In national and international competitions of the International Brazilian Jiu-Jitsu Federation, there are nine weight categories for males (<57.5, 64, 70, 76, 82.3, 88.3, 94.3, 100.5 and <100.5 kg) and eight weight categories for females (<48.5, 53.5, 58.5, 64, 69, 74, 79.3 and <79.3 kg). Brazilian jiu-jitsu competitions are also divided according to athletes’ age as follows: juvenile (15–17 years of age), adult (>18 years of age) and master (>30 years of age) [2]. The duration of matches takes these variables into account and can vary from 5 min for white belts to 10 min for black belts [2].

Athletes start fighting from a standing position, but most of the combat takes place in groundwork [3]. The aim of the sport is to make your opponent give up the combat by means of choke, joint locks (wrist, elbow, knee and ankle locks) or pressure techniques, but when there is no submission the matches are decided by the scoring of specific techniques (takedown, guard pass, mount, back mount, back control, knee on belly and sweep), and in the event of a draw by the referee’s decision [2].

The main characteristic of Brazilian jiu-jitsu is intermittency [4]. An athlete has to perform on average four to six matches to become champion in the main competitions of the modality [1, 4]. Various capacities and physical skills are required during a jiu-jitsu match, and thus, the athletes need to be in excellent physical condition to support the demands of the training and consequently the matches [5]. In this sense, as examples, we can cite aerobic power, which collaborates to maintain a high intensity throughout the match, delay fatigue and achieve a better/faster recovery between matches [6]; muscle strength, which is used for both attack and defence; muscle power, used in the application of throwing techniques or in some specific movements of groundwork actions (sweeps and guard pass); muscular endurance for maintaining grip on the opponent’s gi (specific apparel for training) when there is a gripping dispute, to dominate the opponent and apply techniques and maintain positions; the reaction time used to dodge and/or anticipate the opponent’s attacks or take advantage of opportune moments for the application of attacks; and flexibility, which collaborates in specific situations of attack or defence [1, 7]. Also, due to the fact that athletes are divided according to body mass, the fighters are required to present a low percentage of body fat, with greater muscle development, predominantly a mesomorphic profile, which is associated with competitive success, since athletes often reduce their body mass to compete [8, 9].

Given this dynamic of the matches, the athletes are required to possess a high level of fitness. In this regard, for an organization and training prescription with greater specificity, it is essential to know the physical and physiological profiles of the sport’s athletes. Other combat sports such as judo [10], wrestling [11], amateur boxing [12], taekwondo [13] and karate [14] have had this profile very well described in the literature.

However, to date, there have been no in-depth review papers that synthesize the physical and physiological characteristics of Brazilian jiu-jitsu athletes. A review of Brazilian jiu-jitsu athletes’ characteristics could improve the knowledge of coaches, and strength and conditioning of trainers, concerning the physical and physiological profiles needed to reach a high level of performance in this combat sport. Thus, the aim of the present study is to provide a comprehensive review that will help scientists, coaches and athletes to better understand the physical and physiological profile requirements of Brazilian jiu-jitsu. Finally, it is important to point out that the current systematic review centred on analysing the studies involving the gi or kimono in Brazilian jiu-jitsu athletes.

## Methods

### Searches

A computer literature search was carried out of the PubMed, ISI Web of Knowledge, SportDiscus and Scopus databases (up to January 2016) for English-language, peer-reviewed articles. The keywords used were as follows: “Brazilian jiu-jitsu”, “Brazilian jiu-jitsu” AND “performance” OR “physical fitness” OR “physiology” OR “body composition” OR “somatotype” OR “aerobic fitness” OR “anaerobic fitness” OR “strength” OR “muscle power” OR “muscular endurance” OR “flexibility” OR “reaction time” OR “speed” OR “agility”. Articles published in English, Portuguese or Spanish were included. References (articles, books and congress abstracts) from the original studies were searched for further relevant investigations.

This systematic review is reported in accordance with Meta-analysis Of Observational Studies in Epidemiology (MOOSE) [15].

### Study Inclusion and Exclusion Criteria

Only scientific researches dealing with the major fitness components of Brazilian jiu-jitsu athletes (body composition and somatotype, aerobic and anaerobic profiles, muscular strength, muscle power, muscle endurance, flexibility, reaction time, speed and agility) and using accepted methods that provided relevant practical applications for a Brazilian jiu-jitsu athlete’s fitness training and/or performance were included in the current review. Besides, just studies involving athletes (male and female) were added. However, one study involving practitioners was added due to high number of subjects [16].

### Assessment of Risk of Bias

For the present systematic review, two reviewers independently analysed the titles and abstracts of the articles retrieved from the literature search and reviewed the full text of the published articles. Any disagreements between the reviewers regarding study inclusion were resolved by a third investigator.

## Results

### Search Results

**Fig. 1:**
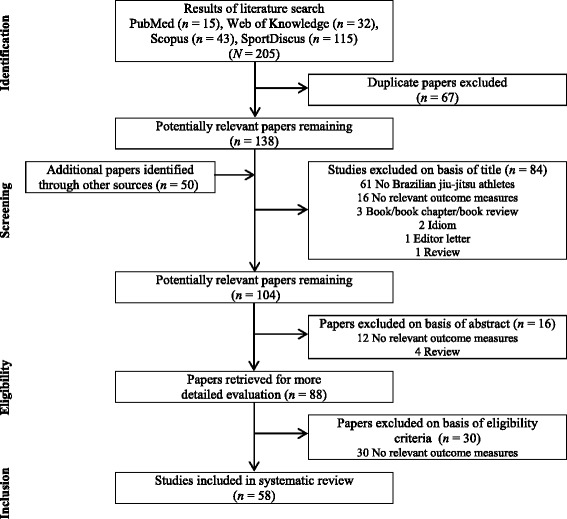
Flow chart illustrating the different phases of the search and study selection

Figure 1 shows a flow chart summarizing the results of the systematic search that identified a total of 205 searches in the electronic databases PubMed, Web of Knowledge, Scopus and SportDiscus. After having added relevant studies from other sources (e.g. reference lists from original and review articles) and after having screened the articles by title, removed duplicates and excluded ineligible articles, 58 studies remained and were included in the present study. A total of 1496 subjects participated in the 58 studies.

## Discussion

### Body Composition

It is crucial to know an athlete’s body composition in combat sport modalities in order to control and define the weight category. In the adult category, the weight classes range from <48.5 to >84.3 kg with kimono for females, and <57.5 to >100.5 kg with kimono for male athletes [2]. Thus, it is impracticable to establish a fat percentage profile for all weight classes. In addition, in other combat sports such as judo, a higher body fat percentage is negatively correlated with performance in locomotion and technical entrance activities [17, 18].

In the present study (Table 1), a higher variation in the body fat percentage was observed, ranging from 5.3 to 19.9% for male athletes. However, the average values observed were of ~12%. Only two studies were found involving female athletes and the results showed average values of 19.3 and 24.2% body fat percentage. It is worth emphasizing that the protocols used in order to estimate the percentage of body fat in Brazilian jiu-jitsu athletes were generic, or may exhibit variation in the method selected; therefore, comparisons between athletes of different studies should be made with caution.

**Table 1:** Body fat percentage of Brazilian jiu-jitsu athletes (data are presented as the mean ± SD)

Athlete characteristics (*n*)	Body mass (kg)	Body fat (%)	Method (Prediction equation reference)	References
Male				
High level athletes (*n* = 14)	71.3 ± 9.1	8.5 ± 1.5	Carter [84]	Diaz-Lara et al. [85]
High level athletes (*n* = 8)	76 ± 10	11.2 ± 3.0^a^	Lohman [86]	Marinho et al. [25]
State level athletes (*n* = 10)	72 ± 5	14.8 ± 3.0		
Athletes of different competitive levels—brown and black belt (*n* = 10)	81.8 ± 7.4	13.0 ± 4.8	Jackson and Pollock [87]	Andreato et al. [39]
High level athletes (*n* = 26)	75.4 ± 9.7	9.5 ± 2.1	Bioimpedance—Moon et al. [88]	Diaz-Lara et al. [89]
State level athletes—blue to black belt (*n* = 15)	82.2 ± 11.9	14.6 ± 5.6	Jackson and Pollock [87]	Follmer et al. [90]
Athletes of different competitive levels (*n* = 9)	73.3 ± 9.7	6.8 ± 2.1	Jackson and Pollock [87]	Mazzoccante et al. [35]
State level athletes (*n* = 9)	77.4 ± 24.5	16.5 ± 7.1	Jackson and Pollock [87]	Ribeiro et al. [36]
State level athletes (*n* = 9)	84.6 ± 19.9	16.1 ± 5.6		
Practitioners (*n* = 136)	81.8 ± 13.1	16.2 ± 6.7	Jackson and Pollock [87]	Schwartz et al. [16]
High level athletes—brown and black belt^b^	77.9 ± 6.8	7.4 ± 2.7	Jackson and Pollock [87]	Silva et al. [68]
Practitioners—blue and purple belt^b^	76.5 ± 8.7	6.5 ± 1.7		
High level athletes—purple to black belt			Kerr [91]	Báez et al. [26]
Pass fighter (*n* = 10)	75.0 ± 8.9	18.4 ± 2.5		
Guard fighter (*n* = 15)	75.9 ± 11.9	19.9 ± 2.5^c^		
All (*n* = 25)	75.6 ± 10.6	19.3 ± 2.5		
High level athletes (*n* = 20)	81.6 ± 5.4	7.1 ± 5.4	NR	Brandão et al. [22]
Practitioners (*n* = 10)	83.3 ± 3.8	7.8 ± 2.5	NR	
State level athletes (*n* = 20)	68.1 + 8.5	12.0 + 4.3	Jackson and Pollock [87]	Carmo et al. [92]
High level athletes			Bioimpedance—Moon et al. [88]	Diaz-Lara et al. [20]
Novice—white to blue belt (*n* = 24)	75.7 ± 9.3	9.3 ± 3.7		
Expert—purple to black belt (*n* = 32)	77.4 ± 11.3	9.1 ± 4.6		
Brown or black belt (*n* = 10)	83.8 ± 12.1	16.1 ± 10.1	Jackson and Pollock [87]	Joel et al. [52]
Brown or black belt (*n* = 10)	83.1 ± 10.1	16.6 ± 8.0		
Practitioners—men (*n* = 8)	78.9 ± 7.9	19.4 ± 5.2	NR	Lorenço-Lima et al. [93]
Athletes of different competitive levels (*n* = 10)	70.3 ± 5.9	6.8 ± 2.4	Jackson and Pollock [87]	Mazzoccante et al. [37]
High level athletes (*n* = 49)	77.3 ± 6.5	15.7 ± 4.0	Bioimpedance	Pietraszewska et al. [94]
High level athletes (*n* = 10)	70.0 ± 3.3	14.1 ± 2.9	Thorland et al. [95]	Pinho-Júnior et al. [96]
Practitioners—purple to black (NR)	78.7 ± 7.9	9.5 ± 1.9	NR	Tinsley et al. [97]
High level athletes (*n* = 11)	83.1 ± 8.7	10.3 ± 2.6	Jackson and Pollock [87]	Andreato et al. [8]
Athletes of different competitive levels (*n* = 12)	75.4 ± 8.8	9.2 ± 2.4	Jackson and Pollock [87]	Andreato et al. [98]
Athletes of different competitive levels			Tipton and Oppliger [99]	Arruda et al. [99]
Feather (*n* = 3)	69.8^d^	10.5^d^		
Light (*n* = 1)	47.0	5.3		
Middle (*n* = 3)	81.6^d^	9.8^d^		
Medium heavy (*n* = 4)	87.6^d^	12.5^d^		
Heavy (*n* = 1)	90.0	14.6		
Ultra heavy (*n* = 2)	95.0^d^	14.5^d^		
All (*n* = 14)	82.8 ± 9.1	11.4 ± 3.6		
Athletes of different competitive levels (*n* = 155)	78.2 ± 8.5	15.9 ± 6.1	Thorland et al. [95]	Brito et al. [9]
High level athletes (*n* = 9)	68.0 ± 2.8	12.7 ± 3.5	Thorland et al. [95]	Santos et al. [100]
State level athletes (*n* = 21)	78.1 ± 9.4	14.3 ± 4.8	NR	Souza-Junior et al. [101]
Beginner athletes—white and blue belt (*n* = 7)	74.9 ± 5.9	8.7 ± 3.8	Jackson and Pollock [87]	Coswig et al. [21]
Experienced athletes—more than purple belt (*n* = 7)	79.0 ± 4.1	9.1 ± 3.0	Jackson and Pollock [87]	
High level athletes (*n* = 7)	77.9 ± 3.5	9.0 ± 2.6	Jackson and Pollock [87]	Andreato et al. [23]
State level athletes (*n* = 7)	72.2 ± 25.0	9.1 ± 3.1	Jackson and Pollock [87]	
High level athletes (*n* = 8)	79.4 ± 9.5	8.4 ± 2.3	Jackson and Pollock [87]	Gomes et al. [24]
State level athletes (*n* = 5)	72.4 ± 7.7	9.8 ± 2.4	Jackson and Pollock [87]	
High level athletes (*n* = 106)	76.0 ± 15.2	15.8 ± 6.1	Thorland et al. [95]	Roas et al. [102]
State level athletes—purple to black belt (*n* = 5)	93.8 ± 13.7	19.9 ± 4.3	Jackson and Pollock [87]	Rigatto [103]
State level athletes—purple to black belt (*n* = 7)	80.4 ± 16.6	12.2 ± 5.7		
High level athletes (*n* = 7)	78.9 ± 12.2	9.8 ± 4.2	Guedes and Guedes [104]	Del Vecchio et al. [3]
Female				
Practitioners (*n* = 8)	56.3 ± 10.0	24.2 ± 3.6		Lorenço-Lima et al. [93]
Athletes (*n* = 14)	61.0 ± 11.5	19.3 ± 6.3	Slaughter et al. [105]	Roas et al. [102]

*NR* not reported

Pass fighter: athlete fighting passing the guard (rather than playing above)

Guard fighter: athlete fighting inside the guard (prefers to fight underneath)

^a^Significant difference from non-elite group from the same study

^b^Did not report the number for each group, in total the study included 28 subjects

^c^Significant difference versus pass fighter group from the same study

^d^Only mean value

In judo, it has been observed that there are gradual increases in body fat with the increase in the weight class [10]. In Brazilian jiu-jitsu, this phenomenon also seems to occur, despite the small number of subjects in the single study that showed the body fat values of athletes from different categories [19].

Two studies found no differences between novice and expert athletes when comparing body composition [20, 21]. When comparing elite and non-elite athletes, three studies [22–24] found no differences, but one study [25] found differences in body composition, with a lower body fat percentage for the elite group. However, an interesting fact observed was that the fighting style may require different biotypes, since a pass fighter had a lower percentage of fat than a guard fighter [26]. In this sense, research that considers the association among body fat percentage, technical actions and fighting style (passer and guard player) may be relevant for the training prescription of Brazilian jiu-jitsu athletes.

### Somatotype

It has been suggested that somatotype and sports success are positively correlated. In grappling sports (e.g. judo, wrestling and Brazilian jiu-jitsu), the mesomorphy component has been highlighted as the most relevant for performance [8, 10, 11], and one that also allows discrimination of athletes from different performance levels [27, 28].

The systematic search indicated that only four studies examined the somatotype of Brazilian jiu-jitsu athletes (Table 2). However, in all of them the mesomorphic component was predominant (range 5.5 to 7.9), a fact that coincides with previous studies that evaluated body composition, indicating a good muscle development of the athletes in other grappling sports [10, 11]. In addition, in one of these studies [26] the athletes were divided based on their fighting style and it was observed that pass fighters showed higher values of mesomorphy and lower values of ectomorphy than guard fighters. Based on empirical observations, these results were to be expected, since it has been observed that there is a greater use of strength in guard pass work, and that athletes with ectomorphic characteristics tend to have more facility in performing guard works. However, further studies in this direction are needed to confirm these findings.

**Table 2:** Somatotype of Brazilian jiu-jitsu athletes (data are presented as the mean ± SD)

Athlete characteristics (*n*)	Endomorphy	Mesomorphy	Ectomorphy	Reference
Male				
High level athletes				Báez et al. [26]
Pass fighter (*n* = 10)	2.3 ± 0.6	7.0 ± 1.2	1.3 ± 0.6	
Guard fighter (*n* = 15)	2.2 ± 0.7	5.9 ± 0. 9 ^a^	2.1 ± 0. 9 ª	
All (*n* = 25)	2.2 ± 0.7	6.3 ± 1.1	1.8 ± 0.9	
High level athletes (*n* = 49)	2.1 ± 0.6	5.8 ± 1.0	2.0 ± 0.8	Pietraszewska et al. [94]
High level athletes (*n* = 11)	3.0 ± 0.8	5.5 ± 1.0	1.7 ± 0.6	Andreato et al. [8]
High level athletes (*n* = 7)	3.2 ± 1.6	7.9 ± 1.4	1.7 ± 0.6	Del Vecchio et al. [3]

^a^Significant difference versus pass fighter group from the same study

### Aerobic Profile

In combat sports, high levels of aerobic power and capacity allow athletes to maintain a high intensity throughout the match, contribute to sustaining effort for the entire combat duration and help in achieving a better/faster recovery between matches [6, 29, 30].

For aerobic power, VO_2max_ values were observed between 42 and 52 mL/kg/min in males. In fact, there are still a few studies that have investigated the aerobic profile in Brazilian jiu-jitsu athletes. Only four studies employed the gold-standard method, i.e. direct spirometry to analyse maximal oxygen uptake (generic means to measure the VO_2max_) [31–34]. However, those studies measured VO_2max_ during treadmill tests, a condition that does not occur during matches. The remaining studies (six in total) utilized indirect methods to predict the athletes’ VO_2max_ [1, 16, 35–38]. Additionally, no studies have been found that investigated the aerobic profile of female athletes in Brazilian jiu-jitsu. Brazilian jiu-jitsu is a combat sport, characterized by intermittency, due to the performance of high-intensity efforts interspersed with rest periods [39]. During the match, fluctuations are observed in effort intensity; however, this intensity is very difficult to measure, as the actions performed during the match depend on technical and tactical movements, the opponents and the fighting style [3, 40]. Indeed, the aerobic contribution is predominant in other combat sports, such as karate [41, 42], taekwondo [43, 44] and boxing [45]. Nevertheless, no studies have been found that investigated aerobic fitness during Brazilian jiu-jitsu matches. The measurement of the energy system’s contribution during a Brazilian jiu-jitsu match seems to present a challenge for sport science researchers. This is because it is difficult to assess the specific pathways in the fight demand, given that the fight involves guard passing techniques, sweeps, takedowns, back control, submissions, and direct contact with the ground and the opponent [39]. The development of specific tests would be important to estimate the physiological demand of Brazilian jiu-jitsu matches, as tests for guarder and passer athletes could help coaches to adjust their training prescriptions.

Maximal efforts performed with a duration of more than 75 s show the predominance of the aerobic contribution [46], and high-intensity interval training has been shown to be a good method for improving aerobic and anaerobic fitness for the general population, as well as for athletes from several sports modalities [47]. In combat sports, few studies have investigated high-intensity interval training [36, 47–51]. Only Ribeiro et al. [36] used specific actions/movements of Brazilian jiu-jitsu; the other studies employed generic means, such as running, for improving aerobic and anaerobic fitness. However, the study by Ribeiro et al. [52] has some limitations, such as the use of indirect tests to estimate VO_2max_ and a lack of statistical comparisons between groups. They only used the effect size to verify that high-intensity interval training was better than the usual Brazilian jiu-jitsu training.

An analysis of the table (Table 3) shows that aerobic fitness is not able to discriminate the performance of Brazilian jiu-jitsu athletes of different competitive levels (in accordance with the statistical analysis). These results are in line with other combat sports, such as judo [10]. However, the development of aerobic fitness can be relevant to the recovery between/during matches [10]. This would be important because Brazilian jiu-jitsu athletes perform several matches during one day of competition [2]. Finally, it would be interesting to carry out tests to estimate VO_2max_ on a cycle ergometer for upper and lower limbs as these responses could help coaches in the prescription of generic and specific training.

**Table 3:** Aerobic fitness of Brazilian jiu-jitsu athletes (data are presented as the mean ± SD)

Athlete characteristics (*n*)	Test	VO_2max_ (ml/kg/min)	References
State level athletes (*n* = 5)	Graded exercise test in treadmill (direct)	45.2 ± 2.4	Leitão da Silva [31]
Athletes of different competitive levels (*n* = 9)	1600 m test	50.4 ± 4.0	Mazzoccante et al. [35]^a^
State level athletes—white to purple belt (*n* = 9)	2400 m test	42.4 ± 5.6	Ribeiro et al. [36]
State level athletes—white to purple belt (*n* = 9)		46.3 ± 7.0	
State level athletes (*n* = 9)		46.3 ± 7.0	
Practitioners (*n* = 136)	Queens College step	52.2 ± 7.9	Schwartz et al. [16]
Athletes of different competitive levels (*n* = 10)	1600 m test	50.2 ± 4.3	Mazzoccante et al. [37]^b^
State level athletes—purple to black belt (*n* = 14)	1600 m test	49.0 ± 3.2	Silva et al. [38]
State level athletes—white belt (*n* = 14)		51.0 ± 3.6	
High level athletes (*n* = 7)	Graded exercise test in treadmill (direct)	42.7 ± 3.2	Rezende et al. [32]
State level athletes (*n* = 8)	Graded exercise test in treadmill (direct)	49.8 ± 2.3	Borges et al. [33]^c^
Practitioners (*n* = 30)	Graded exercise test in treadmill (direct)	52.0 ± 6.9	Mazzocante et al. [34]
	1600 m test	52.1 ± 5.1	
High level athletes (*n* = 10)	Graded exercise test in treadmill (indirect)	49.4 ± 3.6	Vidal-Andreato et al. [1]

VO_2max:_ aerobic power

Direct method_:_ using gas analyser

Indirect method: means by validated formulas

^a^12.4 ± 1.3 km/h for the anaerobic threshold velocity

^b^12.3 ± 1.5 km/h for the anaerobic threshold velocity

^c^41.7 ± 2.0 ml/kg/min at anaerobic threshold

### Anaerobic Profile

This topic is not a review of the glycolytic anaerobic system. However, to enable better understanding, it was decided to include some information concerning this system, to provide a greater insight into the energetic demand of Brazilian jiu-jitsu matches. Anaerobic capacity and anaerobic power are widely involved in different combat sports because the decisive moments in these sports involve great energy demands, which cannot be supplied solely by oxidative metabolism [10]. The Wingate test has often been used to estimate anaerobic performance in the combat sports domain, such as for judo and wrestling [10, 53]. Although the Wingate test is a generic test and cannot be classified as a gold-standard measure, the test presents a large sensibility in the different phases of sports training periodization [53]. Furthermore, the maintenance of high-intensity efforts is associated with anaerobic power and capacity, i.e. power refers to the peak, while capacity refers to the average during 30 s of the Wingate test [10]. Del Vecchio et al. [3] performed a Wingate test for lower limbs on Brazilian jiu-jitsu high-level athletes (*n* = 7), with 7.5% of the body mass of the athletes, and observed the following values for the variables analysed: peak power (10.1 ± 1.2 W/kg), mean power (9.9 ± 1.4 W/kg) and fatigue index (48.2 ± 9.4%), respectively. In another study, Leitão da Silva [31] reported the following values for the Wingate test on lower limbs in Brazilian jiu-jitsu athletes (*n* = 5), also using 7.5% of the body mass: peak power (11.5 ± 1.4 W/kg), mean power (9.8 ± 0.4 W/kg) and fatigue index (56.5 ± 11.0%), respectively.

In fact, anaerobic power and anaerobic capacity represent the ability to generate and maintain a high-intensity performance over seconds that can be extended up to a few minutes [54]. Moreover, anaerobic capacity and anaerobic power are associated with maintaining an intermittent performance of high intensity, in which the decisive sports actions are dependent on movement and powerful actions [55, 56]. The values obtained for peak power and mean power from Brazilian jiu-jitsu athletes are higher than those considered excellent for healthy people [53]. However, the elaboration of an anaerobic profile of Brazilian jiu-jitsu athletes is limited by low number of studies. Only two studies reporting the anaerobic responses in the Wingate test for lower limbs were found, and the anaerobic responses in the Wingate test to upper limbs are still unknown.

Based on the aspects listed above, the lack of studies that have investigated the anaerobic responses in lower limbs and the absence of studies focusing on upper limbs are noteworthy. Nevertheless, recently a specific jiu-jitsu anaerobic performance test was proposed that required performance in an adapted protocol (i.e. Brazilian jiu-jitsu technique). The authors found a correlation between high lactate concentrations and heart rate values measured during simulated Brazilian jiu-jitsu combat and the specific jiu-jitsu anaerobic performance test [57]. Thus, the realization of adapted protocols for the measurement of high-intensity intermittent performance (for example: four sets × 1 min execution with 45 s recovery time) can assist coaches in their training prescriptions aimed at maintaining high-intensity intermittent performance [57].

### Maximum Isometric Handgrip Strength

Maximum isometric strength is characterized by actions that do not alter the muscle length, i.e. there is no movement of the joint, and thus, it is static [58, 59]. In grappling combat sports, such as Brazilian jiu-jitsu, judo and wrestling, there is a great demand for maximal isometric handgrip strength [60]. For elite or experienced Brazilian jiu-jitsu athletes, the different studies showed maximum isometric handgrip strength values of between 48 and 57 kg force (kgf) (Table 4). Grip endurance seems to be an important factor for success in immobilizations, takedowns, throws and submissions. It is worth highlighting as a possible limitation that these adjustments generated by isometric training are angle-dependent; thus, the adaptations occur at the angles in which the stimuli occur [59]. One study shows differences between maximum isometric handgrip strength of the right and left hands, in which the right hand had higher values than the left hand in Brazilian jiu-jitsu athletes [61].

**Table 4:** Isometric handgrip strength (IHGS) of Brazilian jiu-jitsu athletes (data are presented as the mean ± SD)

Athlete characteristics (*n*)	Right IHGS (kgf)	Left IHGS (kgf)	References
Male			
High level athletes (*n* = 14)	53.5 ± 3.2^a^	48.5 ± 5.2^b^	Diaz-Lara et al. [106]
State level athletes—brown and black belt (*n* = 10)	53 ± 6^a^	50 ± 9^b^	Andreato et al. [4]
High level athletes (*n* = 26)	48.5 ± 5.6^a^	50.8 ± 5.2^b^	Diaz-Lara et al. [89]
State level athletes (*n* = 22)	54.2 ± 6.7^c^	51.4 ± 6.1	Franchini et al. [72]
State level athletes—blue to black belt (*n* = 15)	48.1 ± 5.7	45.5 ± 7.9	Follmer et al. [90]
State level athletes^d^			Gasparotto et al. [107]
White belt (*n* = 18)	40.1 ± 2.5^e^		
Blue belt (*n* = 15)	46.3 ± 3.3^e^		
Purple belt (*n* = 20)	41.4 ± 3.5^e^		
Practitioners (*n* = 136)	103 ± 17^f^		Schwartz et al. [16]
State level athletes—blue belt (*n* = 12)	38.0 ± 6.3^a^	32.3 ± 6.3^b^	Andreato et al. [108]
High level athletes			Diaz-Lara et al. [20]
Novice—white to blue belt (*n* = 24)	43.6 ± 7.1^g^	43.3 ± 6.6^g^	
Expert—purple to black belt (*n* = 32)	48.6 ± 6. 1	49.1 ± 7.0	
High level athletes (*n* = 49)	47.8 ± 8.3	46.2 ± 7.6	Pietraszewska et al. [94]
Experienced—purple to black (*n* = 14)	52.4 ± 11.8	50.6 ± 11.6	Silva et al. [38]
Beginners—white belt (*n* = 14)	46.7 ± 6.5	47.2 ± 5.8	
State level athletes—white to brown belt (*n* = 35)	45.9 ± 10.3	44.2 ± 11.1	Andreato et al. [40]
State level athletes			Costa and Oliveira [109]
White and blue belt (*n* = 29)	43.6 ± 9.0	41.1 ± 7.2	
Purple to black belt (*n* = 17)	46.3 ± 8.9	45.1 ± 9.6	
All (*n* = 46)	43.8 ± 10.2	42.3 ± 9.6	
Beginner athletes—white and blue belt (*n* = 7)	52.5 ± 9.1	49.9 ± 7.2	Coswig et al. [21]
Experienced athletes—more than purple belt and high level (*n* = 7)	57.0 ± 8.4	55.6 ± 7.6	
High level (*n* = 11)	43.7 ± 4.8	40.1 ± 3.8	Vidal-Andreato et al. [1]
State level athletes—white and blue belt (*n* = 5)	33.6 ± 5.5^a^		Neto and Dechechi, [110]
High level athletes^d^			Matuzaki et al. [69]
White belt (*n* = 15)	42.3 ± 10.2^e^		
Blue belt (*n* = 17)	49.2 ± 11.3^e^		
Black belt (*n* = 6)	50.2 ± 5.2^e^		
High level athletes—brown and black belt (*n* = 21)	51.2 ± 10.7	48.2 ± 10.3	Oliveira et al. [62]
High level athletes—blue and purple belt (*n* = 29)	49.6 ± 8.2	46.2 ± 8.2	
All (*n* = 50)	50.3 ± 9.1	47.0 ± 9.0	

*Kgf* kilogramme force

^a^Dominant handgrip

^b^Non-dominant handgrip

^c^Significant difference versus left handgrip from the same study

^d^There was no comparison between groups

^e^Did not report the side evaluated

^f^Sum of both hands

^g^Significant difference versus expert group from the same study

### Dynamic Strength

The data available in the scientific literature are limited to maximum dynamic strength in Brazilian jiu-jitsu athletes, and a few studies have investigated these responses in these athletes; furthermore, there are no data available in the scientific literature for maximum strength in female Brazilian jiu-jitsu athletes. Thus, more research needs to be conducted on this topic. One-repetition-maximum (1RM) tests have been widely used for strength training prescription [62]. Maximal dynamic strength can be associated with sporting excellence or success in Brazilian jiu-jitsu athletes because studies have shown that 1RM performance for the bench press was greater in advanced or elite than in non-advanced or non-elite athletes [25, 31] (Table 5). In Olympic wrestlers, these same differences between different level groups are also observed for the bench press exercise and are also found in lower limbs (e.g. squat exercise) [63].

**Table 5:** One-repetition-maximum (1RM) data in different exercises performed by Brazilian jiu-jitsu athletes (data are presented as the mean ± SD)

Athlete characteristics (*n*)	Strength test	Absolute 1RM score (kg)	Relative 1RM score (kg/body mass)	References
Male				
High level athletes (*n* = 8)	Bench press	111 ± 6^a^	1.46 ± 0.13	Marinho et al. [25]
State level athletes (*n* = 10)		98 ± 6	1.36 ± 0.11	
High level athletes (*n* = 14)		90.5 ± 7.7	1.27 ± 0.27	Diaz-Lara et al. [106]
High level athletes—brown and black belt^b^		115 ± 16^c^	1.48 ± 0.15^c^	Leitão da Silva et al. [31]
Intermediary athletes—blue belt and purple^b^		101 ± 13	1.32 ± 0.14	
High level athletes—blue to black belt (*n* = 23)		103.4 ± 22.9	1.3 ± 0.2	Silva et al. [111]
State level athletes (*n* = 20)		85.8 ± 17.8	NR	Costa et al. [112]
State level athletes—purple to black belt (*n* = 5)		94 ± 24	NR	Rigatto [103]
State level athletes—purple to black belt (*n* = 7)		87 ± 31	NR	
High level athletes (*n* = 7)		109 ± 18	1.39 ± 0.26	Del Vecchio et al. [3]
High level athletes (*n* = 8)	Squat	91 ± 8	1.20 ± 0.13	Marinho et al. [25]
State level athletes (*n* = 10)		88 ± 7	1.23 ± 0.13	
High level athletes (*n* = 7)		110 ± 15	1.38 ± 0.19	Del Vecchio et al. [3]
State level athletes—purple to black belt (*n* = 5)	Lat pull-down	90 ± 18	NR	Rigatto [103]
State level athletes—purple to black belt (*n* = 7)		86 ± 15	NR	
State level athletes—purple to black belt (*n* = 5)	Military press	56 ± 17	NR	Rigatto [103]
State level athletes—purple to black belt (*n* = 7)		57 ± 22	NR	
High level athletes—(*n* = 11)	Leg Press	308 ± 88	NR	Fernandes et al. [113]
High level athletes (*n* = 7)	Deadlift	138 ± 24	1.72 ± 0.31	Del Vecchio et al. [3]
State level athletes—purple to black belt (*n* = 5)	Biceps curl	91 ± 25	NR	Rigatto [103]
State level athletes—purple to black belt (*n* = 7)		52 ± 29	NR	
State level athletes—purple to black belt (*n* = 5)	Elbow extension in the pulley	114 ± 28	NR	Rigatto [103]
State level athletes—purple to black belt (*n* = 7)		64 ± 41	NR	

*NR* not reported

^a^Significant difference versus non-elite group from the same study

^b^Did not report the number for each group, in total the study included 28 subjects

^c^Significant difference versus non-advanced group from the same study

Moreover, strength training plays an important role in training periodization, because it serves as the basis for the other periodization phases, such as strength endurance and muscle power (Table 6) [64]. It is worth mentioning that strength training must emphasize the main muscles related to the movements and actions performed during the matches. Hypertrophy training may be harmful to the athlete who is weighing in near the upper limit of their category because the hypertrophy will provide weight gain [65]. In addition, maximum strength training can be an important tool for athletes, since there will be an increase in this capacity and not muscular hypertrophy [66]. In this regard, the specific literature gives to sport scientists and coaches some interesting values in relation to relative 1RM score both for upper and lower limbs in Brazilian jiu-jitsu elite athletes (i.e. bench press 1.3–1.5 kg/body mass, squat 1.2 kg/body mass and deadlift 1.7 kg/body mass) (Table 4). Knowledge of these results is important for drawing comparisons.

**Table 6:** Peak torque (N m kg^−1^), power (W) or total work (J) during isokinetic maximal tests performed by Brazilian jiu-jitsu athletes (data are presented as the mean ± SD)

Athlete characteristics (*n*)	Exercise	Result	References
Male			
State level athletes—blue to black belt (*n* = 15)	Elbow flexion		Follmer et al. [90]
	PT_45°_ (N m kg^−1^)	0.77 ± 0.12	
	PT_90°_ (N m kg^−1^)	0.91 ± 0.16	
	PT_120°_ (N m kg^−1^)	0.77 ± 0.13	
	PT_CON_ (N m kg^−1^)	0.68 ± 0.17	
	PT_ECC_ (N m kg^−1^)	0.87 ± 0.20	
	Elbow extension		
	PT_45°_ (N m kg^−1^)	0.63 ± 0.10	
	PT_90°_ (N m kg^−1^)	0.71 ± 0.13	
	PT_120°_ (N m kg^−1^)	0.71 ± 0.13	
	PT_CON_ (N m kg^−1^)	0.79 ± 0.19	
	PT_ECC_ (N m kg^−1^)	1.10 ± 0.25	
State level athletes (*n* = 15)	Knee flexion		Assis et al. [115]
	PT (N/m)	187^a^	
	Power (W)	252	
	Total work (J)	1913	
	Knee extension		
	PT (N/m)	308^a^	
	Power (W)	286^a^	
	Total work (J)	1614	

*PT* peak torque, *PT*_*CON*_ peak torque concentric, *PT*_*ECC*_^:^ peak torque eccentric

^a^Only mean value

Similarly, a few studies have described the maximum torque in Brazilian jiu-jitsu athletes. This test (isokinetic) has been used extensively to measure muscle imbalances between the different sides of the body and the antagonistic muscles. Usually, this test is indicated after surgery and injuries during rehabilitation; moreover, values equal to or less than 5% for the different limbs are considered ideal after rehabilitation for returning to sports training [67].

### Muscle Power

Several authors explain that the decisive moments that determine the result of a match (guard passes, sweeps, takedowns etc.) require muscular power actions [34, 68, 69]. Jump performance can be a factor that discriminates between two groups with different levels of training and experience in Brazilian jiu-jitsu [20]. Recent studies have found that Brazilian jiu-jitsu athletes scored high results in the CMJ, which ranged between 30 and 45 cm (Table 7), which was higher than data from Olympic wrestlers [70] and similar to that from senior top elite judo athletes [71]. The vertical jump height showed no gradual decrease during the simulated competition (i.e. four matches of 10 min) [4].

**Table 7:** Muscle power in Brazilian jiu-jitsu athletes (data are presented as the mean ± SD)

Athlete characteristics (*n*)	Exercise	Result (cm)	References
Male			
State level athletes (*n* = 10)	Throw the medicine ball	428 ± 33	Nascimento [73]
State level athletes—white and blue belt (*n* = 5)		380 ± 48	Neto and Dechechi [110]
High level athletes (*n* = 14)	Countermovement jump	40.6 ± 2.6	Diaz-Lara et al. [106]
State level athletes—brown and black belt (*n* = 10)		41 ± 6	Andreato et al. [4]
High level athletes (*n* = 26)		34.0 ± 5.2	Diaz-Lara et al. [89]
State level athletes			Diaz-Lara et al. [20]
Novice—white to blue belt (*n* = 24)		29.7 ± 5.0^a^	
Expert—purple to black belt (*n* = 32)		34.2 ± 5.1	
State level athletes—blue to black belt (*n* = 23)		40.8 ± 5.5	Silva et al. [111]
State level athletes—blue to purple belt (*n* = 22)		45.5 ± 1.3	Detanico et al. [114]
State level athletes (*n* = 9)^b^	Vertical Jump	39.9 ± 8.1	Ribeiro et al. [36]
State level athletes (*n* = 9)^b^		48.3 ± 5.9	
High level athletes (*n* = 49)	Standing long jump	234 ± 22	Pietraszewska et al. [94]
Beginner athletes—white and blue belt (*n* = 7)		225 ± 25	Coswig et al. [21]
Experienced athletes—more than purple belt and national level (*n* = 7)		226 ± 12	
State level athletes (*n* = 10)		237 ± 23	Nascimento [73]
State level athletes—white and blue belt (*n* = 5)	Standing long jump	234 ± 25	Neto and Dechechi, [110]

^a^Significant difference versus expert group from the same study

^b^The study included two different groups with athletes with the same characteristics

However, when the bench press throwing exercise was used to determine peak power, no difference was found with loads of 1RM between 30 and 60% between advanced and non-advanced Brazilian jiu-jitsu competitors [68]. Lastly, there are two research studies that analysed the load that optimized muscle power output in Brazilian jiu-jitsu [68] in the bench press throw exercise (~42% of 1RM) and [72] in the bench press (45.1 ± 12.9% of 1RM). Slightly lower results in the bench press exercise were observed for wrestlers, who obtained their maximal power production at 34–37% of 1RM.

Based on the results specified above, it can be concluded that decisive actions and therefore athletic performance during Brazilian jiu-jitsu matches are mainly dependent on muscular power, both in upper and lower limbs.

### Muscular Endurance

Muscular endurance is the ability of a muscle or a group of muscles to sustain repeated contractions against resistance for an extended period [73]. The Brazilian jiu-jitsu athlete during a match is in contact with the opponent most of the time and must maintain a strong grip on different body parts [69]. Thus, due to this dynamic, the most gripping actions performed in Brazilian jiu-jitsu require high resistance to maintain constant levels of strength endurance for a long time. In addition, there is a consensus in the specific literature on the importance of grip strength endurance or gripping endurance [1, 34, 74]. Corroborating this assertion, Andreato et al. [4] revealed that Brazilian jiu-jitsu athletes in competitive situations reported higher perceptions of fatigue in the forearm region (68%). Thus, the training of this region should be covered in the training programme.

There are two specific tests that evaluate gripping endurance, one statically: maximum static lift (grip endurance with gi or kimono) and one dynamically: maximum dynamic lift (chin-up repetitions with gi or kimono) [75–78]. Mean grip endurance performances reported for national and international competitors in the literature ranged from 54 to 62 s (Table 8). These grip endurance performances are higher than those generated by elite judo athletes at 35 ± 18 s [75], whereas the repetitions with kimono performances ranged between 15 and 18 reps (Table 8), which was also slightly higher than elite judo athletes at 12 ± 5 reps [76]. However, Franchini et al. [77] indicated that state-level athletes had similar values to elite athletes in *judogi* chin-up repetitions (isometric and dynamic endurance strength). Therefore, the development of studies that elaborate normative tables with a large sample size may be relevant for classifying Brazilian jiu-jitsu athletes.

**Table 8:** Muscular endurance in different exercises performed by Brazilian jiu-jitsu athletes (data are presented as the mean ± SD)

Athlete characteristics (*n*)	Exercise	Result (s or rep.)	References
Male			
High level athletes (*n* = 14)	Grip endurance with gi	54.4 ± 13.4 s	Diaz-Lara et al. [106]
Athletes—brown and black belt (*n* = 8)		28 ± 9 s	Andreato et al. [4]
Athletes—blue to black belt (*n* = 15)		41 ± 16 s	Follmer et al. [90]
High level athletes (*n* = 49)		40 ± 11 s	Pietraszewska et al. [94]
High level athletes (*n* = 10)		61 ± 19 s	Pinho-Júnior et al. [96]
Experienced—purple to black (*n* = 14)		45 ± 10 s^a^	Silva et al. [38]
Beginners—white belt (*n* = 14)		36 ± 10 s	
Athletes of different competitive levels (*n* = 10)		62 ± 14 s^b,c^	Silva et al. [78]
Athletes of different competitive levels (*n* = 10)		60 ± 0 s^b,c^	
Practitioners (*n* = 10)		43 ± 5 s^c^	
Beginners—white belt (*n* = 10)		28 ± 9 s	
High level athletes (*n* = 9)		63 ± 19 s	Santos et al. [100]
High level athletes (*n* = 10)		56 ± 11 s^d^	Silva et al. [75]
State level athletes (*n* = 10)		38 ± 11 s	
Athletes—blue to black belt (*n* = 15)	Repetitions with gi	10 ± 5 rep	Follmer et al. [90]
High level athletes (*n* = 10)		15 ± 2 rep	Pinho-Júnior et al. [96]
Experienced—purple to black (*n* = 14)		10 ± 3 rep	Silva et al. [38]
Beginners—white belt (*n* = 14)		8 ± 3 rep	
High level athletes (*n* = 10)		18 ± 3 rep^b,c^	Silva et al. [78]
State level athletes (*n* = 10)		17 ± 3 rep^b,c^	
Practitioners (*n* = 10)		9 ± 3 rep	
Beginners—white belt (*n* = 10)		7 ± 3 rep	
High level athletes (*n* = 9)		15 ± 2 rep	Santos et al. [100]
High level athletes (*n* = 10)		15 ± 4 rep^d^	Silva et al. [75]
State level athletes (*n* = 10)		8 ± 3 rep	
Beginner athletes—white and blue belt (*n* = 7)		10 ± 3 rep^e^	Coswig et al. [21]
Experienced athletes—more than purple belt and national level (*n* = 7)		15 ± 2 rep	
High level athletes (*n* = 14)	Bench press at 45% 1 RM	22 ± 8 rep	Diaz Lara et al. [106]
High level athletes (*n* = 8)	Push-ups	41 ± 3 rep^f^	Marinho et al. [25]
State level athletes (*n* = 10)		36 ± 3 rep	
High level athletes (*n* = 11)		39 ± 8 rep	Vidal-Andreato et al. [1]
High level athletes (*n* = 8)	Sit-ups	46 ± 4 rep^f^	Marinho et al. [25]
State level athletes (*n* = 10)		40 ± 3 rep	
State level athletes (*n* = 9)		52 ± 19 rep	Ribeiro et al. [36]
State level athletes (*n* = 9)		68 ± 11 rep	
Practitioners (*n* = 136)		62 ± 16 rep	Schwartz et al. [16]
High level athletes (*n* = 49)		35 ± 5 rep	Pietraszewska et al. [94]
High level athletes (*n* = 11)		52 ± 7 rep	Vidal-Andreato et al. [1]

^a^Significant difference versus beginners group from the same study

^b^Significant difference versus recreational group from the same study

^c^Significant difference versus beginners group from the same study

^d^Significant difference versus non-elite group from the same study

^e^Significant difference versus experienced group from the same study

^f^Significant difference versus non-elite group from the same study

Both tests can discriminate among athletes with different levels and experience in Brazilian jiu-jitsu [38, 75, 78]. However, the permanence of static isometric grip strength could be a completely specific and individualized manifestation for Brazilian jiu-jitsu due to the major permanence of grip holding during Brazilian jiu-jitsu matches versus other grappling sports [3].

It is common in combat sports to evaluate muscular endurance using sit-ups and push-ups. Brazilian jiu-jitsu elite athletes were rated as excellent for abdominal and upper limb strength endurance [1]. The results in sit-ups (Table 7) are similar to those for elite junior judo athletes [79] and international medallists in taekwondo [13]. In the push-ups test, lower results are observed compared to other similar elite athletes both in judo [80] and wrestling [80]. From all the results presented above, it is possible to conclude that muscle endurance is one of the most critical components of Brazilian jiu-jitsu performance, for many reasons: athletes need to have high abdominal strength endurance and maintain a strong grip for an extended amount of time [1, 2, 4, 69], and additionally, they must repeat muscular power actions during the development of combat or as match duration increases [60].

### Flexibility

Flexibility is a relevant physical component of Brazilian jiu-jitsu, specifically in the thoracolumbar spine and hamstrings, which are required to perform specific situations of attack or defence [1, 7]. A high level of flexibility can help Brazilian jiu-jitsu athletes to perform positions. In addition, good flexibility can facilitate the learning of these motor gestures [1]. However, there are no specific tests for evaluating the flexibility of Brazilian jiu-jitsu athletes, and therefore, it is necessary to develop tests for such purposes. Table 9 shows the results on the flexibility of Brazilian jiu-jitsu athletes in the sit-and-reach test.

**Table 9:** Flexibility of Brazilian jiu-jitsu athletes as measured by the sit-and-reach test (data are presented as mean ± SD)

Athlete characteristics (*n*)	Sit-and-reach (cm)	Reference
Male		
High level athletes (*n* = 8)	40 ± 3^a^	Marinho et al. [25]
State level athletes (*n* = 10)	32 ± 3	
Athletes—brown and black belt (*n* = 10)	26 ± 8	Andreato et al. [4]
Practitioners (*n* = 136)	27 ± 9	Schwartz et al. [16]
Athletes—adult (*n* = 30)	22 ± 8	Araujo et al. [81]
Athletes—master (*n* = 7)	26 ± 7	
Athletes—senior (*n* = 4)	22 ± 6	
Beginner athletes—white and blue belt (*n* = 7)	28 ± 2^b^	Coswig et al. [21]
Experienced athletes—more than purple belt and national level (*n* = 7)	35 ± 4	
High level athletes (*n* = 11)	35 ± 8	Vidal-Andreato et al. [1]
State level athletes (*n* = 20)	34 ± 7	
High level athletes (*n* = 7)	43 ± 3	Del Vecchio et al. [3]

^a^Significant difference versus non-elite group from the same study

^b^Significant difference versus experienced group from the same study

For flexibility, evaluated by the sit-and-reach test, there were wide-ranging results, with variations of 22 cm in adults and senior fighters [81] and up to 43 cm in high-performance athletes [3]. Thus experience and competitive level seem to influence an athlete’s flexibility responses, as experienced athletes had greater flexibility than beginner athletes [21] and elite athletes showed more flexibility than non-elite athletes [25]. Therefore, further studies are needed to confirm the idea that the competitive level can have an influence on flexibility. This idea is plausible, since these results have already been seen in wrestlers, with high-level athletes showing greater flexibility than low-level athletes [11].

### Reaction Time

Success in open skills seems to be determined by the capacity of an individual to adapt his behaviour to changes imposed by their opponent. Often, this adaptation has to be extremely fast [82]. In combat sports, reaction speed is important for athletes to dodge and/or anticipate their opponent’s attacks or take advantage of opportune moments for their own attacks [4, 7]. Thus, reaction speed can be crucial in defining matches, in addition to being one of the factors that could explain a drop in performance during a competition. However, despite the importance of this variable, only two studies have evaluated it. In the first study, reaction time (the time it took for the athlete to identify the beep sound; in other words, the first movement in the contact pad) was evaluated, and a value of 239 ± 17 ms was found in practitioners of the modality (*n* = 11). Moreover, older individuals (27–35 years old; *n* = 3; 248 ± 14 ms) did not differ from younger individuals (18–26 years old; *n* = 8; 236 ± 17 ms) [83]. In the second study, the response time (the time that athletes take to make a jump after identifying the beep sound) was evaluated and a mean value of 0.40 ± 0.04 s was observed in brown and black belt athletes (*n* = 9) [4]. It is important to note that there is a lack of studies evaluating the reaction time in Brazilian jiu-jitsu. Moreover, there are serious limitations in interpreting the results because the test used to evaluate the reaction time is very generic, and the same test is used in different sports.

## Conclusions

In general, Brazilian jiu-jitsu athletes had low body fat, without differences between novices and experts or between elite and non-elite athletes. The mesomorphic component was predominant. Aerobic power was similar to that of other grappling combat sports and did not seem to be influenced by the Brazilian jiu-jitsu athlete’s competitive level. Further research is needed to quantify anaerobic power, especially in upper limbs. The values of isometric handgrip strength are not high. However, specific tests for grip strength endurance using the gi can discriminate athletes with different experience and competitive levels in Brazilian jiu-jitsu. More studies are necessary to describe the maximal strength profile of Brazilian jiu-jitsu athletes. However, until now, maximal dynamic strength has been associated with sporting excellence or success in Brazilian jiu-jitsu athletes in upper limbs. Decisive actions and therefore athletic performance during Brazilian jiu-jitsu matches are mainly dependent on muscular power in both upper and lower limbs; however, more studies are necessary to describe the power strength profile of Brazilian jiu-jitsu athletes. With regard to flexibility, experience and competitive level seem to influence an athlete’s flexibility responses, as experienced athletes had greater flexibility than beginners and elite athletes showed more flexibility than non-elite athletes. Lastly, more research is required to find out whether reaction time can be a determining factor in athletic success in Brazilian jiu-jitsu.

Thus, based on the aspects described above, it is remarkable to note that there are a few studies mapping the performance of Brazilian jiu-jitsu athletes, especially involving variables such as aerobic and anaerobic power and aerobic and anaerobic capacity. Because of the intermittent characteristic of Brazilian jiu-jitsu, it is important to carry out more research to report the anaerobic power and capacity performance of such athletes. Moreover, longitudinal studies describing the responses of Brazilian jiu-jitsu athletes to physical training and competitive performance are incipient. Thus, new studies with this purpose are indispensable. Researches describing the female sex are also indispensable, given that only two studies have investigated this population, in which only body composition was measured. In addition, further research is needed to analyse whether there are differences between sex, belt ranks, competitive level and sport experience time, and among the different weight categories for different variables.

### Practical Applications

There is no doubt that the practice of Brazilian jiu-jitsu has grown exponentially all over the world in the last few decades. As a consequence of this growth, researchers have strived to enhance the quality of investigations on physical and physiological responses to training, combat simulation, competition and official competitions in Brazilian jiu-jitsu. However, there is an urgent need for a better understanding of the referred aspects in order to provide ideal training prescriptions. The available scientific literature on these issues needs to be reinforced, and doubts need to be settled with regard to physical and physiological responses in Brazilian jiu-jitsu.

Additionally, studies seeking to investigate the effects of resistance training for improved performance of flexor and extensor forearm muscles can be relevant for sustaining grip during Brazilian jiu-jitsu matches, given that grip power provides a more effective form of controlling the adversary. Moreover, scientific studies carried out on strength training with different time spans, i.e. linear, undulating, block and complex training, as well as Olympic weightlifting, can be tested on Brazilian jiu-jitsu athletes, with the objective of improving performance in competition. Similarly, there is an urgent need to carry out studies that investigate the effects of protocols that develop flexibility among Brazilian jiu-jitsu athletes, given that many of the “guard fighter” athletes’ positions require high flexibility of the torso and lower limbs, thereby facilitating sweeps, back control and the consequent defeat of the opponent.

Finally, it is evident that scientific studies with athletes at international level are scarce, especially among female athletes. As a result of these findings, it is understood that there is an urgent need to carry out comprehensive studies with athletes of different age groups (adults, masters and seniors), different grades (white, blue, purple, brown and black), different weight categories (feather to heavy, as well as absolute), with or without gi (with or without kimono), as well as among male and female athletes. Furthermore, studies that focus on the above-mentioned aspects will provide highly significant indicators for directing Brazilian jiu-jitsu training.

## Abbreviations

1RM: One-repetition maximum
IHGS: Isometric handgrip strength
MOOSE: Meta-analysis Of Observational Studies in Epidemiology
NR: Not reported
PT: Peak torque
PT_CON_: Peak torque concentric
PT_ECC_: Peak torque eccentric
VO_2max_: Aerobic power
